# Predictors of alcohol consumption among in-school adolescents in the Central Region of Ghana: A baseline information for developing cognitive-behavioural interventions

**DOI:** 10.1371/journal.pone.0207093

**Published:** 2018-11-12

**Authors:** Thomas Hormenu, John Elvis Hagan Jnr, Thomas Schack

**Affiliations:** 1 Department of Health, Physical Education and Recreation, University of Cape Coast, Cape Coast, Ghana; 2 "Neurocognition and Action—Biomechanics"- Research Group, Faculty of Psychology and Sport Sciences, Bielefeld University, Bielefeld, Germany; 3 Center of Excellence "Cognitive Interaction Technology" CITEC, Bielefeld University, Bielefeld, Germany; Stellenbosch University, SOUTH AFRICA

## Abstract

**Background and purpose:**

Despite a recent shift in school going adolescents’ engagement in health compromising behaviours and their related socio-economic implications on developing societies, it is surprising that baseline information for planned interventions is sparse. The purpose of this study was to investigate the prevalence of alcohol drinking and related behaviours among in-school adolescents in the Junior High Schools (JHS) in the Central Region of Ghana.

**Methods and results:**

Descriptive cross-sectional design was employed with multistage sampling procedures to sample 1400 school going adolescents in JHS in the Central Region. Preliminary findings using simple frequencies and percentages revealed 42% alcohol drinking prevalence in the region. High prevalence of drunkenness (73%, n = 406) and early exposure to alcohol drinking when students were in primary school (52%, n = 286) were noted. Community festivals and use of alcohol as a form of medicine were enabling factors of alcohol consumption in the region. Binary logistic regression analysis also showed that geographical location was a significant predictor of alcohol drinking among school going adolescents, with students in the southern and central part of the region at greater risks of drinking alcohol than those from the northern part (OR = .696, 95% CI = 0.52–926, *p* = .013). However, no statistical significant variations were found in the odds of drinking alcohol for age (OR = 1.13, 95% CI = 0.86–1.48, *p* = .370), gender (OR = .81, 95% CI = 0.65–1.01, *p* = .06), religious affiliation (OR = 1.33, 95% CI = 0.94–1.89, *p* = .10), parental communication (OR = .86, 95% CI = 0.66–1.06, *p* = .13), academic performance (OR = 1.07, 95% CI = 0.79–1.45, *p* = .05) and socioeconomic status (OR = 1.20, 95% CI = 0.95–1.53, *p* = .12).

**Conclusions:**

With this baseline data, it was recommended that schools’ curricula should include preventive cognitive-behavioural interventions that teach drug resistance skills and anti-drug norms. These interventions would foster the development of requisite knowledge and social skills (e.g., developing competence) for resisting social and peer influences that may trigger alcohol use and perhaps other drugs. Potentially, the motivation for alcohol use among school going adolescents in the region would be minimized, if not prevented.

## Introduction

Adolescence has been viewed as the most critical period of human life where health is one of the essential requirements for proper adolescent development [1]. Defining adolescence is cumbersome but Sayer and Patton [2] describe adolescence as the developmental stage between childhood and adulthood. WHO [3] also exemplifies adolescence as the developmental stage, a period between 10 to 19 years. A more comprehensive definition is Driessnack’s [4], who described adolescence as “the psychosocial, emotional, cognitive, and moral transition from childhood to young adulthood” [p. 502]. This time of life, according to Steinberg [5] represents a great share of the foundation for future health and behavioural patterns (i.e., physiological, psychologically and behaviourally). Steinberg further expatiated that adolescent period is key for the adoption of health behaviours relating to drug use and alcohol consumption. This period is characterized by heightened physical growth and rapid changes in height, weight, body shape, and genital development leading to many life experimentations [6]. The desire during this period for most adolescents is the quest to fulfill basic physiological and psychological needs, including social acknowledgment or endorsements and other risks taking experiences like alcohol consumption, other drugs and sexual satisfactions [7]. According to Sayer and Patton [2], this period could be described as a period of *Sturm und Drang* (storm and stress) characterized by conflict with parents, mood disruptions, and risk behaviours [8]. This storm and stress have the potential to throw the health of adolescents off gear leading to the formation of maladjusted behaviours such as anxiety, depression, eating disorders, alcohol consumption and obesity. To Sayer and Patton [2], alcohol drinking experimentations and its associated risky behaviours are very prominent at this stage.

Since the adolescent stage is full of experimentations, varied considerable experiences are gained through existential conflicts such as exposure and vulnerability to substance abuse, particularly alcohol use [7]. Moreover, early initiation of alcohol use is one of the most predictors of future health, socio-cultural and economic problems [9]. A wide range of factors that are considered as facilitators of alcohol use among adolescents are lifestyle, high levels of stress and anxiety, low self-esteem, depressive symptoms, susceptibility to peer pressure and problems associated with school [10, 11]. Even in small amounts, alcohol use has a number of consequences such as risky sexual behaviours, increased suicide rate, violence, juvenile delinquency, familial conflicts, conflicts with friends, a greater risk of accidents and illicit drug use are consequences that have serious implications for public health [10,12,13].

The patterns of alcohol usage in Ghana among school going adolescents show gradual increase in the trends over the years. A study conducted by Adu-Mireku [14] to assess the prevalence and association between alcohol, cigarette, and marijuana use in Ghana among Senior Secondary School students in Accra with 894 sampled students revealed 56.9% girls and 43.1% boys, with mean age = 17.4 years. Using a modified version of the Youth Risk Behaviour Survey questionnaire, overall lifetime alcohol use prevalence of 25.1% was reported. Among lifetime users, current alcohol use was 46.2%. Boys were significantly more likely than girls to be lifetime users of alcohol, but not as current users. In another study in the same year, Dogbe [15] investigated substance use pattern among students and recorded higher prevalence of substance use among students in the secondary school. The Findings in Dogbe’s study revealed that substances most often used included alcohol (85%), reflecting an increased prevalence over the previous years. Furthermore, 49% of participants were introduced to drugs by friends who were not students whereas 17.6% were initiated into alcohol use by student friends. Additionally, 16.2% had their drinking experience through parents.

Other studies have also reported higher alcohol prevalence than what Adu-Mireku found in 2003 but lower than Dogbe’s findings that showed a lifetime 41.6% alcohol prevalence among school going adolescents aged 12–17 years. For example, Assabil [16] found that 51% of students who consumed alcohol were males, although females constituted 45% of the adolescent population in selected second cycle schools. Likewise, Ghana’s lifetime alcohol prevalence due to socioeconomic differences among Ghanaian adolescents aged between 12–18 years in a school-based cross-sectional survey stood at 39.3% [13]. GSHS [17] also reported 78.9% alcohol drinking prevalence among JHS 1–3 students aged 13–17 years in GSHS with 1,648 samples. The GSHS report further indicated that, current drinking prevalence among the studied sample within the past 30 days was 15.8%, with those who were drunk so much that they could not walk prevalence pegged at 9.9%.

Many precursors have been associated with alcohol and other drug related problems across adolescents and college students [1]. This risk based approach research is aimed at developing primary interventions to alcohol use, misuse and abuse by school going adolescents and other problem behaviours [17]. There is scholarly evidence that diverse precursors such as age, gender, academics, peers, religion, geographical location, and parental attachment influence drinking among school adolescents [18, 19, 20, 21]. Quite a number of reviews have indicated that age of first use of alcohol is highly associated to both persistent use and rate of use [22,23,24–27]. For instance, the younger a child initiates alcohol and drug abuse, the higher the risk of serious health implications and later adult substance abuse [25,26]. Notably, children who are initiated into alcohol before the age of 6 years are more than twice as likely to report frequent, heavy or problematic drinking at age 15 compared to children who are not exposed before the age of 13 years [28]. However, other research found that the age of first drink neither predicted alcohol use by age 20 nor the consequences from alcohol abuse by age 30 [29] and that early drinking was only a modest predictor of heavy regular drinking in later life [30]. Some studies have also reported findings related to gender influences [31,32]. Some research evidence show that variations in alcohol use between males and females are rather converging. However, it appears there is more convergence on some alcohol indicators than others [31]. According to Johnston, O’Malley and Bachman [32], male and female 8th and 10th graders had similar rates of both using alcohol and having been drunk in the previous 30 days. Nevertheless, gender differences emerged among 12th graders, with males having a higher 30-day prevalence of alcohol use and of having been drunk. These authors found equal prevalence of lifetime alcohol use for boys and girls at each grade level. However, a clear gender variance showed in binge drinking in the previous 2 weeks and on daily use of alcohol among the 12th graders. Johnson and co-workers concluded that, although alcohol use and risky drinking practices (e.g., getting drunk, binge drinking) occur among both boys and girls, these practices are more prevalent among boys by the 12th grade.

One variable that has been associated with alcohol use among adolescent students is academic attainment or performance [33]. Loveland-Cherry [33] noted that school attachment and receiving considerable good grades have been related to less adolescents’ alcohol use, whereas poor grades and absenteeism were linked to early initiation and increased levels of alcohol use. Similarly, male and female students who drank more than 5 and four drinks in a session, one or two times in a 2 week period were over three times more likely to fall behind in academic work compared with more moderate drinkers. When the drinking frequency increased to 3 times in a 2 week period, these adolescents were more than eight times more likely to report this academic relapse [34]. However, other evidence claims that the association between academic performance and college drinking is inconclusive due to methodological limitations of research that examined the association between alcohol and academic performance [35]. In addition, poor parental practices like less communication and low degree of bonding or connectedness to their adolescent children appear to increase the risk for problem behaviours including misuse and abuse of alcohol and other drugs [36]. Other studies have confirmed a positive relationship between alcohol use and family environment [37–39]. Similarly, students who were noted with family related issues and met alcohol misuse criteria were recorded as having a significantly greater history of lifetime substance use and were more likely to use other drugs like marijuana [40]. On other factors, developing societies with socioeconomic disadvantages such as poverty, poor housing, and over-crowding have been shown to be associated with an increased risk of childhood/adolescents’ problems and delinquency [41]. However, other research on social class and drug use has not always confirmed these popular stereotypes [42]. According to Hawkins, Catalano and Miller [42], only when poverty is extreme and occurs in conjunction with childhood behavioural problems has it shown to increase the risk for later alcoholism and other drug related problems in adolescents.

Evidence from these research reports indicates that alcohol consumption among adolescents has become a general societal problem and is on the increase, especially in developing countries like Ghana. To date, there seems to be no evidence of such increases in the central region in the country. Due to the complex and ever-changing developmental profile of adolescents, there is the need to keep up-to-date data on their health related behaviours for regular multifaceted oriented interventions to ensure healthy developments of this target population. Good timing of developmental psychologists and health promoters of puberty and subsequent guidance would lead to the development of appropriate health promoting intervention programmes in relation to alcohol usage. According to World Health Organisation [43] since adolescent stage is characterized by many turmoil and uncertainties, regular profiling of health behaviours among adolescents for differentiation is required. Unfortunately, there is inadequate research literature in the Central Region of Ghana on the extent of differentiation in alcohol use among in-school adolescents. The schools may offer an ecologically valid setting and thus provide a convenient and efficient means of reaching this target group before they develop habitual drinking behaviour. This differentiation research may also provide a more distinct picture on the dramatically changing health profile across school going adolescents and also enable greater national and international comparison of trends associated with alcohol use. This study therefore sought to investigate the prevalence and other drinking related behaviours as well as their predictors among in-school adolescents in some selected schools in the Central Region. Based on previous research [18–33, 41–42], it was hypothesized that all selected socio-demographic variables would significantly predict alcohol drinking behaviour among the sampled in-school adolescents.

## Materials and methods

### Participants’ selection

Ethical clearance was approved by University of Cape Coast, Institutional Review Board [UCCIRB/CES/2016/04] after inspecting the study protocol that was without parental consent. This waiver was granted based on evidence that parental permission can be waived off when conducting research on sensitive issues on active mature minors. This approach helps in obtaining real information from the participants because parental and teacher involvements in these research can affect the truthfulness and participants’ participation. Additionally, the capacity to consent is related to the nature and complexity of the research. Therefore, if adolescents are mature enough to understand the purpose of the proposed study and their involvement requested, they are mature enough to consent. Based on these assertions, all study participants signed the inform consent form after being assured of their anonymity and confidentiality at all stages of the data collection process. Participants were assured that information collected was solely for academic purpose and that their participation was entirely voluntary. This meant that study participants could stop with the data collection process at any point that they felt like doing so and that there were no right or wrong answers with their responses.

We used a cross-sectional design approach, with the accessible population consisting of all school going adolescents in the JHS within the age of 10–15 years. Adolescents at this level of education are between 10–15 years and per this study are found in Forms 1–3. The sample was categorized into three groups; 10–11, 12–13, and 14–15 years [44]. However, all the students sampled were above 11 years, hence, analysis was done on two groups; 12–13 and 14–15 years. Sequel to this, a multistage sampling technique involving cluster, simple random and convenience procedures were used to sample 1,400 school going adolescents in the JHS in the Central region of Ghana. This sample size was determined using Cohen ‘G’ power with effect size of .40, confidence level of 95% and confidence interval of .05. However, the return rate 94% (N = 1,311) of the questionnaire were retrieved. Different procedures were employed for the sampling. Stage one involved cluster sampling to put districts of the Region into three geographical zones (southern, central and northern). The clustering made the zones well-defined and helped maintain the homogeneity of the population. At the second stage, purposive sampling procedure was employed to select two districts from each of the zones whereas third stage involved the use of proportionate simple random sampling to select 10% of schools from each of the two districts selected from each of the zones. Convenience sampling method was used to select averagely 40 students from each of the 34 schools, with equal representation of boys and girls at the fifth stage.

### Instrumentation

A modified version of the generic Global School Health Survey [17] questionnaire was adapted to suit the context of this study. This study adapted only the items related alcohol drinking and demographics, hence the modification reduced the items to 15. The items measuring drinking behaviours among school adolescents were used. Variables measured were students’ drinking behaviour using Yes/No, frequency of drinking, drunkenness, educational level of first drinking behaviour, perception of friends drinking, sources of drinking and reasons of alcohol usage. The data collection process took four weeks. Questionnaires were coded and data entered into SPSS Version 22. The internal consistency measure for the instrument used yielded a reliability coefficient of Kuder-Richardson [KR20] formula 0.84, a value that is deemed reasonable and appropriate for reliability analysis. KR-20 was chosen because is a measure reliability suitable for binary variables (i.e. answers that are right or wrong). The scores for KR-20 ranged from 0 to 1, where 0 is no reliability and 1 is perfect reliability. The closer the score is to 1, the more reliable the test [45].

### Procedure

After the selection of schools, the respective head masters/mistresses received a formal notification about the survey and the plan for organizing series of health education, and promotion programmes on health compromising behaviours across Junior/Senior High Schools in the Central Region of Ghana based on available research evidence. To reduce the burden on schools by ensuring minimal disruption of regular school academic work, pre-arranged sessions were staggered across all the selected schools chosen within the sampled districts over a period of three months. On each day of questionnaire administration, one research and a team of trained research assistants met with headmasters and mistresses of each selected school and were introduced to the students prior to survey administration. To avoid any contextual influence, teachers excused themselves prior to the start of the survey administration to enhance privacy and confidentiality. This was followed up with an introductory session to brief study participants on the rational of the study and standard instructions needed for completing the questionnaire. The need for honesty and confidentiality of their responses was emphasized to the students, where each item on the instrument should be considered on its own merit and that there was no right or wrong answer. Students subsequently completed the self-administered questionnaire in a classroom and recorded their responses directly on the questionnaire sheet.

### Data analysis

Data were analyzed in different stages using the Statistical Package for Social Sciences ([SPSS] version 22.0 for Windows). Pre-screening procedures were initially conducted to the accuracy and appropriateness of the data. All statistical assumptions were subsequently tested. Descriptive statistics (simple frequencies and percentages) were employed to determine the prevalence of alcohol drinking behaviours among in-school adolescents in the region. Follow-up binary logistic regression analysis was further used to ascertain the relative influence of the socio-demographic factors on alcohol consumption among the students in the region.

## Results

### Main analysis

#### Prevalence of alcohol consumption

**Fig 1:**
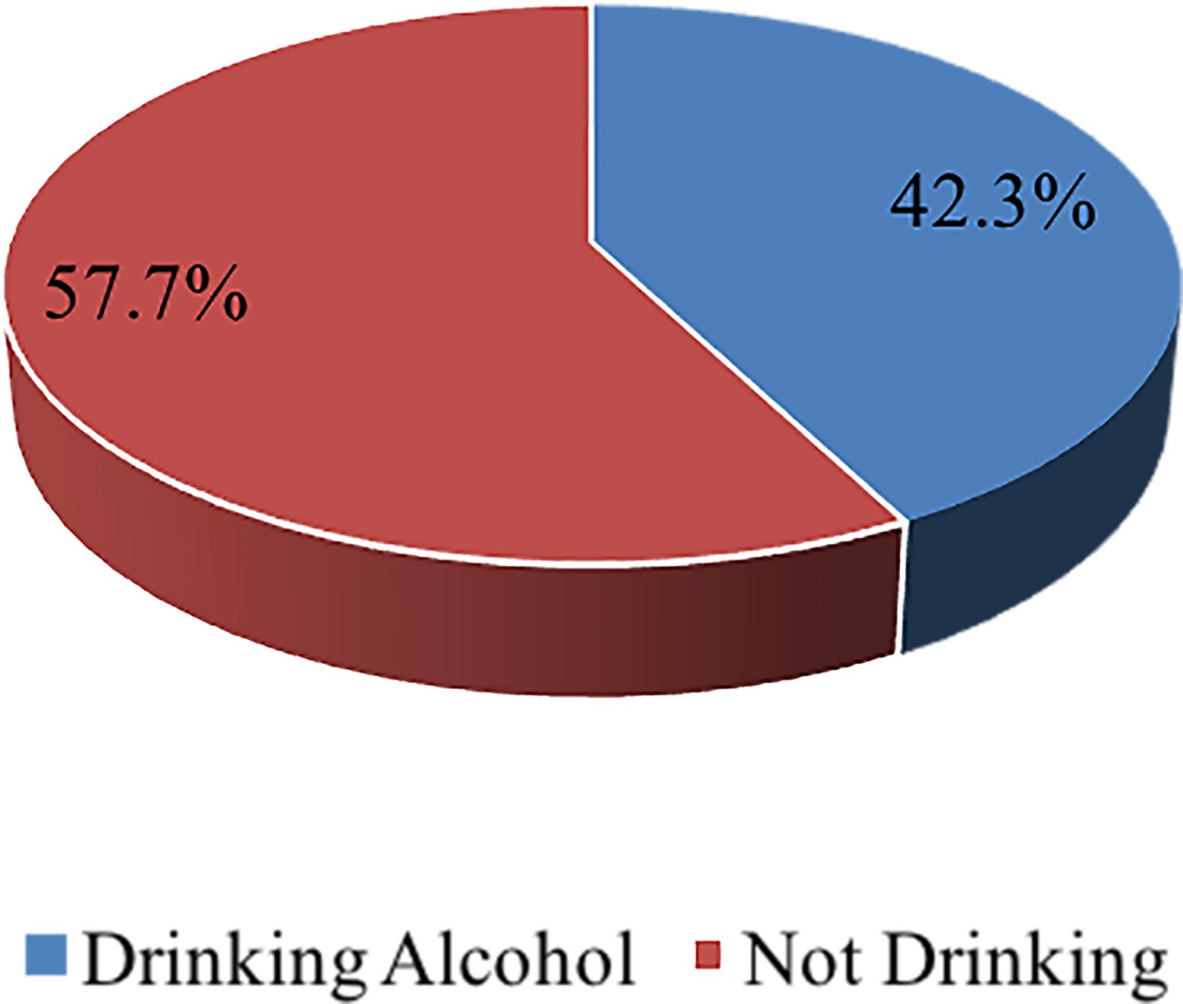
Prevalence of alcohol use among school going adolescents.

Study participants’ responses on alcohol consumption are represented in Fig 1. The results revealed an overall alcohol consumption prevalence of 42% (n = 554) among school going adolescents in the region while 58% (n = 757). Additionally, adolescents who had taken alcohol, 54% (n = 297) had been drunk once, 9% (n = 53) had been drunk twice, and 10% (n = 55) had been drunk several times. These outcomes suggest a high prevalence of drunkenness (73%, n = 406) among school adolescents in the region. Similarly, 24% (n = 133) of alcohol consumption among the sampled students happened at the lower primary school, 28% (n = 154) at the upper primary, and the majority at the JHS 48% (n = 267).

Furthermore, majority of the study participants (45%, n = 247) who had taken alcohol were initiated by friends, 37% (n = 205) parents, and 18% (n = 102) by elders in the community. Key reasons for the onset of alcohol drinking among the school going adolescents are follows: taking alcohol as medicine (48%, n = 268), community festival (20%, n = 109), pleasing friends (20%, n = 108), and 12% (n = 69) wanted to be stronger. To determine how school going adolescents perceived alcohol consumption among their peers, majority 56% (n = 738) noted that none of their peers had been drinking alcohol. However, 44% (n = 573) perceived that some of their friends consumed alcohol. These results are presented in Table 1.

**Table 1:** Drunkeness, sources of alcohol and reasons for alcohol drinking.

Variables	Frequency	Percentage
**Drunkeness**		
Never been Drunk	148	27
Once	297	54
Twice	54	9
Several times	55	10
**Level of Education and Onset of Drinking**		
Lower Primary School	133	24
Upper Primary School	154	28
Junior High School	267	48
**Sources of First Drink of Alcohol**		
Friends	247	45
Parents	205	37
Elders in the Community	102	18
**Reasons for First Alcohol Drinking**		
Taken as Medicine	268	48
Given during Community Festivals	109	20
Taken as Source of Belongingness to my Friends	108	20
In order to stronger	69	12
**Perception of Friends Drinking**		
None of my friends drinks alcohol	738	56
Some of them are drinking alcohol	573	44

#### Predictors of alcohol drinking behaviour

Binary logistic regression results presented in Table 2 revealed that the overall logistic regression model was significant (-2LogL = 1755.6, χ^2^ = 30.210, *p* = .001) as Nagelkerke R^2^ of .031 explains 3.1% of variance on the risk to consume alcohol among school going adolescents. With this percentage contribution to the entire model, the result showed the whole model significantly predicted drinking behaviour among students. In Table 2, statistically significant variations were found in the odds of drinking alcohol with geographical location. School going adolescents in the northern part of the Central Region were less likely to drink alcohol than those in the south (OR = .69, 95% CI = 0.52–0.93, *p =* .013). However, no statistical significant variations were found in the odds of drinking alcohol for age (OR = 1.13, 95% CI = 0.86–1.48, *p =* .370), gender (OR = .81, 95% CI = 0.65–1.01, *p =* .06), religious affiliation (OR = 1.33, 95% CI = 0.94–1.89, *p =* .10), parental communication (OR = .86, 95% CI = 0.66–1.06, *p =* .13), academic performance (OR = 1.07, 95% CI = 0.79–1.45, *p =* .05), and socioeconomic status (OR = 1.20, 95% CI = 0.95–1.53, *p =* .12).

**Table 2:** Binary logistic regression of socio-demographic predictors of alcohol drinking among school going adolescents.

		*Drink*		*Not*	*Drink*	*B*	*Wald*	*OR*	*95%CI*	*Sig*.
		*f*	*%*	*f*	*%*					
**Age**										
12–13 years (ref)		119	39.7	181	60.3					
14–15 years		435	43	576	57	0.123	0.805	1.13	0.86–1.48	0.37
**Gender**										
Boys (ref)		284	45.4	342	55.6					
Girls		270	39.4	415	60.6	-214	3.52	0.808	0.65–1.00	0.061
**Religious Affiliation**										
Christian (ref)		480	41.4	679	58.6					
Muslims		74	48.7	78	51.3	0.288	2.66	1.33	0.94–1.88	0.103
**Parental Com.**										
Difficult (ref)		217	45.9	256	54.1					
Easy		337	40	501	60	-0.179	2.28	0.836	0.66–1.06	0.131
**Socioeconomic**										
Low (ref)		353	40.9	510	59.1					
High		201	44.9	247	55.1	0.186	2.32	1.2	0.95–1.53	0.127
**Academic P**.										
Below average (ref)		110	34.8	141	56.2		5.894			0.052
Average		293	44.4	359	55.6	0.068	0.197	1.07	0.79–1.45	0.657
Above average		151	37	257	63	-0.25	2.126	0.779	0.56–1.09	0.145
**Geo. Location**										
Southern (ref)		199	45	242	55		9.454			.009*
Central		214	45.5	256	54.5	0.045	0.109	1.046	0.80–1.37	0.742
Northern		141	35	259	65	-0.362	6.2	0.696	0.53–0.93	.013*
Constant						-0.148	0.506	0.863		0.477

*Significant results; f = frequency; % = percentage.

## Discussion

The present study sought to examine the prevalence, pattern and predictors of alcohol drinking related behaviours among in-school adolescents in some selected schools in the Central Region of Ghana. Results from the descriptive statistics revealed a lifetime alcohol prevalence of 42% among school going adolescents in the Central Region, a finding that is consistent with Assabil’s [16] findings of 42% alcohol usage among school adolescents aged 12–17 years in the Bosomtwi and Atwima Kwanwoma Districts in the Ashanti Region of Ghana. However, Doku et al. [13] reported 39% lifetime alcohol prevalence among school going adolescents aged 12–18 years from three regions in Ghana. The similarity in the findings could be due to the proposition made by Ralph et al. [46] that transition of adolescents from childhood to adulthood exhibit similar characteristics. However, the prevalence of alcohol consumption among school adolescents in the current study could be described as low compared to the prevalence of 54.3% found by Cofie [47] among JHS students in the Dangme West District of Ghana. The relatively poor socio-economic conditions with high unemployment rate in the Region suggest that many in-school adolescents may have less access to money for the purchase of alcohol and other social enticements [48]. Most people in these deprived areas in the region live on the fringes of life, hence may have other pressing basic needs and priorities than alcohol consumption. Drunkenness was found to be very high among in-school adolescents in the region, thus corroborating previous research [13,46,48]. The plausible reason for this adolescents’ drunkenness might be due to high proliferation of served alcohol at social gatherings; festivals, weddings, and funerals in the region [19]. Additionally, the widespread alluring marketing advertisements of alcoholic drinks and beverages on television, radio, in newspapers, and magazines about their possible ergogenic effects might have accounted for this drunkenness finding [49,50].

Majority of school going adolescents’ first time use of alcohol was in the form of medicine. We imply that there could be possible misconceptions from advertisements in both the print and electronic media on some local alcoholic drinks or beverages popularly termed ‘‘bitters” as treatment drugs for various ailments. These locally manufactured drinks, which are very high on the Ghanaian market are purported to cure all kinds of illnesses such as fever, infertility, hypertension, sexually transmitted diseases as well as improve virility in men, among others. These advertisements have misled the general public. Consequently, it is therefore not surprising that many sexually active adolescents, likewise adults are lured or coaxed into usage due to the perceived effects associated with these alcoholic drinks. According to Ralph et al. [46], alcohol is a depressant, so an early initiation could possibly lead to addiction and physical damage. The influence of friends and parental usage of alcohol as medicine for children were observed to be the point of initial alcohol consumption among school children in the Central region. This finding consolidates the proposition that most young people are introduced to alcohol by their parents at the age of 8–12 years and at 13–19 years by friends [51, 52]. Currie et al. [53] noted that when parents are indifferent, exhibit inappropriate role modelling, or are inconsistent in setting standards of behaviour for their children, there is a much greater likelihood of problem behaviour and psychological problems. Parental usage of alcohol as a form of drug for treatment of illness could be responsible for this high prevalence of alcohol usage among adolescents in the region. The study further revealed early alcohol consumption practice among school adolescents with age of onset at the primary school level (6–12 years). Drinking at home under parental supervision often begins at childhood and may be responsible for the early adolescents’ consumption of alcohol in the current study. Most of these children are exposed and further initiated into drinking due to their involvement in the purchasing of the substance from retail shops in their vicinities by their parents and older family members. On a daily basis, adolescents see their parents drink before and after meals, with friends, and during social gatherings (e.g., naming ceremony, festivals, and funerals). Therefore, the influence of social learning cannot be underestimated in terms of adolescents drinking behaviour.

The hypotheses regarding the predictions on alcohol consumption were partially supported. Specifically, the logistic regression model indicated that geographical location as a factor predicted drinking behaviour among school adolescents in the region. The finding means that students from southern part of the Central Region are at greater risk of drinking alcohol than those from the northern part of the region. This finding is consistent with the previous research findings [20, 21] that geographical location significantly influences alcohol consumption among school adolescents. These authors reported that supportive geographical location such as weather and abundance of alcoholic beverages in the area influence adolescents’ alcohol consumption level. Currie et al. [53] reiterated that engagement in health behaviours patterned geographical differences; hence certain health behaviours depict certain patterns across geographical locations. By inference, adolescents’ easy access to drinking shops in the southern part of the region compared to the northern part due to the rural nature of the districts could be responsible for this difference. Moreover, most adolescents living in the northern part are predominantly rural dwellers and strictly hold on to their traditional African values that may potentially discourage substance use (i.e., alcohol) among this target population [48]. Therefore, adolescents living in these areas are at a higher risk of drinking alcohol than those in the rural parts where there is less number of drinking shops [19]. This outcome means that efforts to control adolescents drinking behaviour should be enforced in the southern part of the region. Bandura’s [54] Social Cognitive Theory (SCT) and Bronfenbrenner’s [55] bioecological model propositions affirm that human behaviours are influenced by environmental predispositions either proximal or distal to the child. Social acceptance of alcohol as a norm for most social gatherings could also be a plausible explanation for alcohol consumption among adolescents in the southern and central parts of the Region. In addition, environmental and social modifications could also be essential tools for eliciting healthy behaviours among adolescents [54,55]. Behaviour modification based on SCT constructs such as self-control, reinforcement, and self-efficacy including goal-setting, self-monitoring and behavioural contracting could be effective interventions for practicing healthy behaviours among school going adolescents in the region [49]. Goal-setting and self-monitoring seem to be particularly useful components of effective interventions. Reciprocal determinism could also be used as an intervention, where those drinking can be both an agent for change and a responder to change. Similarly, role models’ or mentors’ modifications and reinforcements can be used to promote healthier behaviours among school going adolescents in the selected districts.

Nevertheless, age did not predict alcohol usage among school going adolescents in the region. It could possibly be the decrease in the age of onset of alcohol consumption among adolescents [49]. Other studies have reported age as a non-significant factor in alcohol drinking among adolescents but could still be a modest predictor in later years [16]. Although alcohol consumption is socio-culturally reserved to adulthood, it is possible that younger adolescents are consuming alcohol in the region. Nonetheless, other research findings found age as a predictor of alcohol consumption among adolescents with older adolescents at a higher risk of drinking alcohol than the younger counterparts [18].

Gender as factor was also not found to be a predictor of alcohol consumption in the region, thus affirming Cofie’s [47] earlier research results. Although alcohol consumption has been considered as behaviour of masculinity, modernity seems to be narrowing the gap between boys and girls [49]. From this premise, there could be a considerable number of girls drinking alcohol, which according to Cofie [47] could be a precursor for other risky behaviours such as early sexual debut and unwanted pregnancy among these adolescents. However, this finding is in contrast with previous findings that found gender as a significant predictor of drinking behaviour among adolescents with males more likely to drink alcoholic beverages than female adolescents [18, 56, 57, 58]. This difference could be that previous studies were conducted among street connected children, and in the general population with varied heterogeneous characteristics.

Similarly, academic performance did not predict alcohol usage in the current study. This finding contravenes previous studies that found academic performance as a factor associated with alcohol usage among adolescents [59]. However, the finding affirms previous study conducted in Western Europe that academic performance is not a predictor of alcohol drinking behaviour [60]. Sullivan and Wodarski [61] explained that among adolescents, educational performance is not associated with alcohol drinking and that majority of evidence points to the fact that the association between academic performance and drinking is inconclusive. For instance, early exposure to antisocial behaviour in school may predict future alcohol use whereas academic failure in lower classes may trigger early antisocial behaviour or independently contribute to later alcohol use [42]. According to Hawkins, Catalano and Miller [42], what is unclear from existing studies is when could high or low school academic performance become a stable predictor of alcohol use. Therefore, methodological limitations of using the cross sectional survey approach could be responsible and that more research evidence is required particularly with longitudinal studies before the question of whether or not alcohol is associated with academic performance can be answered.

Although previous research reports parental relationship as a significant predictor of drinking behaviour among adolescents such that parental bonding, supervision, strength of communication and adherence to prosocial norms are protective factors in hindering early alcohol consumption [62,63,64], the current research found otherwise. We infer that a possible decrease in the degree of parental connectedness (e.g., communication, bonding) to their children in the region could have accounted for the current finding. Furthermore, socioeconomic status also did not predict alcohol usage in the school going adolescents in the region, a finding that stands in contrast with previous research findings [49]. The previously widened gap between adolescents of low and high socioeconomic status in terms of engagement in deviant behaviours is gradually being narrowed. Another contention is the relative availability of alcohol in small sachets and rubber disposable bottles that can easily be purchased from dotted shops in many parts of the region could be attributed to this outcome. Additionally, many young people growing up in developing societies where alcohol exist in abundance may consequently lead to the increase in risk taking, sensation seeking, and erratic behaviour that follows the onset of puberty [19,46].

Although age, gender, religion, socioeconomic, academic performance and the state of parental communication did not predict alcohol consumption among school going adolescents in the region, the increasing number of school going adolescents’ alcohol drinking behaviour presents a significant challenge for public and school health education in the region. Nevertheless, there are still opportunities that exist to regulate the proliferation and advertisement of alcohol and the utilization of alcohol at public gatherings in the region, especially in the southern and central parts of the region. School health interventions with serious environmental modifications and enforcement of Public Health Act 851 legislations can decrease alcohol drinking and prevent the devastating effects of drinking on health, and educational attainments of these school going adolescents.

### Limitations

This study is limited to some degree by the measurement of the prevalence and predictors of reported alcohol use. The measurement were solely based on adolescents’ self-report, a procedure that is subject to intentional distortion (over or under reporting), memory bias and other social desirability issues inherent with the use of a questionnaire. Despite the relatively homogenous sample in terms of age and schooling (i.e., in-school adolescents aged between 10–15 years), generalizability of the current findings of the study is restricted only to this target population. Future research could consider providing more additional information from peers, parents, and teachers regarding the nature of adolescents’ experiences within the home and school contexts. Again, research on the relationship between school dropout and alcohol consumption is worth studying. Using the longitudinal approach instead of the cross sectional explanations that currently dominate available literature that do not ascribe a causal link would be significant. Also, this study made use of school adolescents and not adolescents in the general population; hence, comparisons of the findings from this cohort with the general adolescents’ population in Ghana should be done with caution. The longitudinal design and the potential adolescent-peer-parent-teacher interactions would better future understanding on school and alcohol use connections in order to deliver pragmatic interventions in the long term. In addition, straight comparison of the current results with other research conducted in other areas of the country (e.g., Accra) should be noted with caution because differences reported might be due to environmental and methodological variations rather than the noted differences *per se* of these studies.

### Practical implications for interventions

Schools’ curricula should include interventions programmes that teach alcohol and other drugs resistance skills and anti-drug norms that would help in the development of personal and social skills. The target is to provide adolescents with the essential knowledge and skills that may help them resist social influences that trigger the use of alcohol and other drugs. These steps might help reduce potential motivations to use these substances by increasing general personal and social competence [65]. According to Botvin [65], schools’ curricula should be designed to address major cognitive-behavioural and psychosocial issues that are either empirically or conceptually related to alcohol and other drug use. Students should be taught cognitive-behavioural skills for building self-esteem, resisting advertisement pressures from the print and electronic media and other pressures from peers, managing anxiety, communicating effectively, developing personal relationships, and asserting one’s rights. These skills ought to be taught using a blend of teaching methods such as focus group discussion, demonstration, modeling, behavioural rehearsal, feedback and reinforcement, and behavioural assignments for out of school practice [66]. Other psychological skills (e.g., goal setting, mental rehearsal, cognitive restructuring) in situations in which adolescents experience direct interpersonal pressure to drink or use other drugs could be taught and practiced through role play. Lastly, information based on the immediate negative implications of alcohol use and decreasing social acceptability of use, and the actual prevalence rates among users could also be emphasized alongside these intervention programmes.

## Conclusion

Although a replication of this study with a wider coverage of in-school adolescents in other parts of the country is required, the present study found 42% prevalence of lifetime alcohol consumption among school going adolescents accompanied by high prevalence of drunkenness in the region. Again, the primary school was identified as the starting point of alcohol consumption, with increased prevalence at JHS. Using alcohol as a medicine to cure diseases and seasonal festivals were the main reasons for alcohol initiation in the region. Further, geographical location was revealed as a significant factor that predicts lifetime alcohol usage among school going adolescents, with students from the northern part of the region at less risk to drink alcohol than those from the southern and central parts of the region. More public health education is needed at the southern and middle part of the region to guide alcohol abusers as well as prevent those about to drink. Parent Teacher Association and School Management Committees should also be educated on the dangers of alcohol to children since most adolescents are initiated into drinking by their own parents. The Food and Drugs Authority in Ghana should enforce Public Health Act 851 which prohibits minors’ access to alcohol and other drugs. Parental support for children and adolescents throughout their development and particularly at critical transition periods where they are most vulnerable (e.g., infancy and early childhood) need critical considerations. Public health education should also focus on both individual and environmental factors that encourage vulnerability and promote resilience.

## Summaries of related operational definitions

A drink constitutes any alcohol consumption amount and tolerance that contribute to an individual’s intoxication level [67]. Moderate drinking is defined as 5 drinks/day and 2 drinks/day whereas heavy drinking, however, is likewise as 5 drinks/day [68]. Similarly, drunkenness as captured in the manuscript refers to the frequency of intoxication where an individual’s ‘bodily functions and mental facilities are in an unmistakably abnormal state’ [69–70].

## Supporting information

S1 Dataset Alcohol drinking behaviour. doi:10.5061/dryad.3g27481.
(SAV)

## References

[1] Awusabo-Asare K, Abane AM, Kumi-Kyereme A. Adolescent sexual and reproductive health in Ghana: A synthesis of research evidence New York, NY: Alan Guttmacher Institute, 2004.

[2] Sawyer S, Patton G. Why adolescent health matters. Health: Future leaders. 2011:110–27.

[3] World Health Organisation (WHO). Young people’s health in context: Selected key findings form the health behaviour in school aged children study. Fact Sheet Euro, vol. 04, pp. 4, 2004.

[4] Driessnack M. Adolescent In Edelman C. L. & Mandle C. L. (Ed.). Health promotion throughout the life span (6th ed). (pp. 502–522) St. Louis: Elsevier Mosby 2006.

[5] Steinberg L. Adolescence (8th ed.). New York: McGraw-Hill 2008.

[6] Hazen E, Schlozman S, Beresin E. Adolescent Psychological. Pediatrics in Review. 2008; 29(5):161 10.1542/pir.29-5-161 18450837

[7] Awabil G, Turkson BA, Bisi O, Badu GK, Bukari N. Adolescent pregnancy in Ghana: Contributing factors, consequences and counselling implications. Ghana Journal Health, Physical Education, Recreation and Dance. 2009; 1(2): 120–133.

[8] Arnett JJ. Adolescent storm and stress, reconsidered. American Psychologist. 1999; 54(5): 317 10354802 10.1037//0003-066x.54.5.317

[9] Strauch ES, Pinheiro RT, Silva RA, Horta BL. Uso de álcool por adolescentes: estudo de base populacional. Revista de Saúde Pública. 2009; 43(4): 647–655. 19618026 10.1590/s0034-89102009005000044

[10] Copeland WE, Angold A, Shanahan L, Dreyfuss J, Dlamini I, Costello EJ. Predicting persistent alcohol problems: a prospective analysis from the Great Smoky Mountain Study. Psychological medicine. 2012; 42(9):1925–35. 10.1017/S0033291711002790 22153225 PMC3411932

[11] Richmond MJ, Mermelstein RJ, Metzger A. Heterogeneous friendship affiliation, problem behaviors, and emotional outcomes among high-risk adolescents. Prevention Science. 2012; 13(3):267–77. 10.1007/s11121-011-0261-2 22207411 PMC3354038

[12] Costa JM, Troncoso ES, Gallego MP, de la Maza VT, Barcenilla AC, Cubells CL, San Molina L. Perfil de los adolescentes que acuden a urgencias por intoxicación enólica aguda. InAnales de pediatría, Elsevier Doyma. 2012; 76(1): 30–37. 10.1016/j.anpedi.2011.07.003 21943507

[13] Doku D, Koivusilta L, Rimpelä A. Socioeconomic differences in alcohol and drug use among Ghanaian adolescents. Addictive Behaviours. 2012; 1:37(3): 357–60. 10.1016/j.addbeh.2011.11.020 22154504

[14] Adu-Mireku S. The prevalence of alcohol, cigarette, and marijuana use among Ghanaian senior secondary students in an urban setting. Journal of Ethnicity in Substance Abuse. 2003; 1:2(1): 53–65.

[15] Dogbe EC. Drug abuse among students of second cycle institutions: a case study of the Tema Secondary School, Masters thesis. 2003.

[16] Assabil JK. Abuse of psychotropic substances-a survey of some first and second cycle institutions in the Bosomtwi and Atwima-Kwanwoma Districts in Ashanti Region of Ghana, Doctoral dissertation. 2010.

[17] Global school-based student health Survey [GSHS]. Global school-based student health Survey: Ghana junior high schools 2012 fact sheet Accra: CDC 2012.

[18] Alvarez-Aguirre A, Alonso-Castillo MM, Zanetti AC. Predictive factors of alcohol and tobacco use in adolescents. Revista latino-americana de enfermagem. 2014; 22(6): 1056–1062. 10.1590/0104-1169.3570.2516 25591103 PMC4309243

[19] Kafuko A, Bukuluki P. Qualitative research in Uganda on Knowledge, attitudes and practices concerning alcohol. Health Communication, YEAH and Afford, Corporate Agreement. 2008(617-A): 00–7.

[20] Patrick ME, Schulenberg JE. Prevalence and predictors of adolescent alcohol use and binge drinking in the United States. Alcohol Research: Current Reviews. 2014; 35(2): 193. 10.35946/arcr.v35.2.10 PMC3908711 24881328

[21] Patrick ME, Schulenberg JE. Alcohol use and heavy episodic drinking prevalence and predictors among national samples of American eighth-and tenth-grade students. Journal of Studies on Alcohol and Drugs. 2010; 71(1): 41–5. 20105412 10.15288/jsad.2010.71.41 PMC2815060

[22] Zeigler D, Wang CC, Yoast RA, Dickinson BD, McCaffree MA, Robinowitz CB, et al The neurocognitive effects of alcohol on adolescents and college students. Preventive Medicine 2005; 40(1): 23–32. 10.1016/j.ypmed.2004.04.044 15530577

[23] Saltz R, Elandt D. College student drinking studies 1976–1985. Contemporary Drug Problems. 1986; 13(1): 117–59.

[24] Donnermeyer J. The use of alcohol, marijuana, and hard drugs by rural adolescents: A review of recent research. Drugs and Society. 1992; 7(1–2): 31–75.

[25] Harolyn M, Belcher HME, Shinitzky HE. Substance abuse in children: Prediction, protection, and prevention. Archives of Pediatrics and Adolescent Medicine. 1998; 152(10): 952–60. 9790604 10.1001/archpedi.152.10.952

[26] Sullivan M, Wodarski J. Rating College Students' Substance Abuse: A Systematic Literature Review. Brief Treatment and Crisis Intervention. 2004; 4(1): 71–91.

[27] Maggs J, Schulenberg JE. Initiation and course of alcohol consumption among adolescents and young adults In Editor GM, ed. Recent developments in alcoholism: Alcohol problems in adolescents and young adults. New York: Kluwer Academic/Plenum Publishers 2005. 10.1007/0-306-48626-1_2 15789858

[28] Bukstein OG. Influences on the risk of and course of substance use and abuse in adolescents. Current Opinion in Psychiatry. 1995; 8(4): 218–21.

[29] Boyd C, McCabe SE, Morales M. College students' alcohol use: A critical review. Annual Review of Nursing Research. 2005; 23: 179–211. 16350766

[30] Saunders B, Baily S. Alcohol and young people: Minimizing the harm. Drug and Alcohol Review. 1993; 12(1): 81–90. 10.1080/09595239300185761 16818315

[31] White HR, Huselid RF. Gender differences in alcohol use during adolescence In Wilsnack RW., & Wilsnack SC. (Eds). Gender and alcohol: Individual and social perspectives. New Brunswick, NJ: Rutgers Center on Alcohol Studies, 1997; 176–198.

[32] Johnston LD, O’Malley PM, Bachman JG. Monitoring the Future National Survey Results on Drug Use, 1975–2002: vol. 1 Secondary school students. (NIH Pub. No. 03–5375). Bethesda, MD: National Institute of Drug Abuse, 2003.

[33] Loveland-Cherry CJ. Alcohol, children, and adolescents. Annual Review of Nursing Research. 2005; 23: 135–77. 16350765

[34] Perkins HW. Surveying the damages: A review of research on consequences of alcohol misuse in college populations. Journal of Studies on Alcohol. 2002(Suppl. 14): 91–100. 10.15288/jsas.2002.s14.91 12022733

[35] Berkowitz A, Perkins HW. Recent research on gender differences in collegiate alcohol use. Journal of American College Health. 1987; 36: 123–9. 10.1080/07448481.1987.9939003 3312362

[36] Brook JS, Brook DW, Gordon AS, Whiteman M, Cohen P. The psychosocial etiology of adolescent drug use: a family interactional approach. Genetic, social, and general psychology monographs. 1990. 2376323

[37] Greenberg JL, Lewis SE, Dodd DK. Overlapping addictions and self-esteem among college men and women. Addictive behaviours. 1999; 24(4): 565–71. 10.1016/s0306-4603(98)00080-x 10466852

[38] Sher KJ, Wood MD, Wood PK, Raskin G. Alcohol outcome expectancies and alcohol use: A latent variable cross-lagged panel study. Journal of Abnormal Psychology. 1996; 105(4): 561 10.1037/0021-843X.105.4.561 8952189

[39] Werner MJ, Walker LS, Greene JW. Concurrent and prospective screening for problem drinking among college students. Journal of Adolescent Health. 1996; 18(4):276–85. 10.1016/1054-139X(95)00207-9 8860792

[40] Bennett ME, Miller JH, Woodall WG. Drinking, binge drinking, and other drug use among southwestern undergraduates: Three-year trends. The American Journal of Drug and Alcohol Abuse. 1999; 25(2): 331–50. 10395164 10.1081/ada-100101864

[41] Farrington DP, Loeber R, Elliott DS, Hawkins JD, Kandel DB, Klein MW, McCord J, Rowe DC, Tremblay RE. Advancing knowledge about the onset of delinquency and crime In Advances in clinical child psychology (pp. 283–342). Springer, Boston, MA 1990.

[42] Hawkins JD, Catalano RF, Miller JY. Risk and protective factors for alcohol and other drug problems in adolescence and early adulthood: implications for substance abuse prevention. Psychological Bulletin. 1992; 112(1): 64 1529040 10.1037/0033-2909.112.1.64

[43] World Health Organisation [WHO]. Adolescent friendly health services: An agenda for change, Geneva: WHO 2002.

[44] Health Behaviour of School Aged Child [HBSC]. Report on the health behaviour in school-aged children (HBSC) study Copenhagen: WHO Regional Office for Europe 2012.

[45] Tabachnick BG, Fidell LS. Using multivariate statistics (5th ed). Boston, MA: Allyn & Bacon, 2007.

[46] DiClemente RJ, Santelli JS, Crosby RA, editors. Adolescent health: Understanding and preventing risk behaviours John Wiley & Sons 2009.

[47] Cofie CN. Prevalence of substance use among junior high school pupils of the Dangme West District. Dissertation is submitted to the University of Ghana for the award of Master of Public Health Degree. 2010.

[48] Madu SN, Matla MQ. Illicit drug use, cigarette smoking and alcohol drinking behaviour among a sample of high school adolescents in the Pietersburg area of the Northern Province, South Africa. Journal of Adolescence. 2003; 26(1): 121–36. 12550825 10.1016/s0140-1971(02)00120-3

[49] Doku D. Substance use and risky sexual behaviours among sexually experienced Ghanaian youth. BMC Public Health. 2012; 12(1): 571 10.1186/1471-2458-12-571 22839700 PMC3517501

[50] Buxton C, Hagan JE. A survey of energy drinks consumption practices among student-athletes in Ghana: Lessons for developing health education intervention programmes. Journal of the International Society of Sports Nutrition. 2012; 9(1): 9 10.1186/1550-2783-9-9 22444601 PMC3331813

[51] Frisher M, Crome I, Macleod J, Bloor R, Hickman M. Predictive factors for illicit drug use among young people: A literature review London: UK Government Publications, 2007.

[52] ter Bogt T, Nic Gabhainn S. Illicit drug use In HBSC research protocol for 2005/06 survey. NY: Scientific rationales for focus areas, 2005.

[53] Currie CE, Elton RA, Todd J, Platt S. Indicators of socioeconomic status for adolescents: The WHO Health Behaviour in School-aged Children Survey. Health Education Research. 1997; 12(3): 385–97. 10174221 10.1093/her/12.3.385

[54] Bandura A. Self-efficacy: Toward a unifying theory of behavioural change. Psychological Review. 1977; 84(2): 191 847061 10.1037//0033-295x.84.2.191

[55] Brofenbrenner U. Toward an experimental ecology of human development. American Psychologist. 1977; 32(7): 513–31.

[56] Ahamad K, DeBeck K, Feng C, Sakakibara T, Kerr T, Wood E. Gender influences on initiation of injecting drug use. The American Journal of Drug and Alcohol Abuse. 2014; 40(2): 151–6. 10.3109/00952990.2013.860983 24405226 PMC4454335

[57] Habtamu D, Adamu A. Assessment of sexual and reproductive health status of street children in Addis Ababa. Journal of Sexually Transmitted Diseases. 2013; 1–20. 10.1155/2013/524076 PMC4437437 26316958

[58] Hadland SE, Marshall BD, Kerr T, Zhang R, Montaner JS, Wood E. A comparison of drug use and risk behaviour profiles among younger and older street youth. Substance use & misuse. 2011; 46(12): 1486–94. 21417557 10.3109/10826084.2011.561516 PMC3799836

[59] Centre for Disease Prevention and Control [CDC]. Health-risk behaviours and academic achievement US: Department of Health and Human Services 2009.

[60] Loveland-Cherry CJ. Alcohol, children, and adolescents. Annual Review of Nursing Research. 2005; 23: 135 16350765

[61] Sullivan M, Wodarski J. Rating college students' substance abuse: A systematic literature review. Brief Treatment and Crisis Intervention. 2004; 4(1): 71.

[62] Kendler KS, Schmitt E, Aggen SH, Prescott CA. Genetic and environmental influences on alcohol, caffeine, cannabis, and nicotine use from early adolescence to middle adulthood. Archives of General Psychiatry. 2008; 65(6): 674–82. 10.1001/archpsyc.65.6.674 18519825 PMC2844891

[63] Fulkerson JA, Pasch KE, Stigler MH, Farbakhsh K, Perry CL, Komro KA. Longitudinal associations between family dinner and adolescent perceptions of parent–child communication among racially diverse urban youth. Journal of Family Psychology. 2010; 24(3): 261 10.1037/a0019311 20545399 PMC2896222

[64] White J, Halliwell E. Alcohol and tobacco use during adolescence: the importance of the family mealtime environment. Journal of Health Psychology. 2010; 15(4): 526–32. 10.1177/1359105309355337 20460409

[65] Botvin GJ. Substance abuse prevention research: Recent developments and future directions. Journal of School Health. 1986; 56(9): 369–74. 3537523 10.1111/j.1746-1561.1986.tb05775.x

[66] Botvin GJ, Griffin KW, Diaz T, Ifill-Williams M. Drug abuse prevention among minority adolescents: Posttest and one-year follow-up of a school-based preventive intervention. Prevention Science. 2001; 2(1): 1–3. 11519371 10.1023/a:1010025311161

[67] Jaccard J, Turrisi R. Cognitive processes and individual differences in judgments relevant to drunk driving. Journal of Personality and Social Psychology 1987;53:135–145 3612486

[68] Abel EL, Kruger ML, Friedl J. How do physicians define “light,”“moderate,” and “heavy” drinking?. Alcoholism: Clinical and Experimental Research. 1998; 22(5):979–84. 10.1111/j.1530-0277.1998.tb03692.x 9726266

[69] Greenberg LA. The definition of an intoxicating beverage. Quarterly journal of studies on alcohol. 1955; 16(2): 316 14385011

[70] Midanik LT. Definitions of drunkenness. Substance use & misuse. 2003; 38(9): 1285–303. 12908812 10.1081/ja-120018485

